# 1-(5-Carboxy­pent­yl)-2,3,3-trimethyl-3*H*-indol-1-ium bromide monohydrate

**DOI:** 10.1107/S1600536809049204

**Published:** 2009-12-16

**Authors:** Angela Winstead, Krystal Hart, Yousef M. Hijji, Jerry P. Jasinski, Ray J. Butcher

**Affiliations:** a Department of Chemistry, Morgan State University, Baltimore, MD 21251, USA; b Department of Chemistry, Keene State College, 229 Main Street, Keene, NH 03435-2001, USA; c Department of Chemistry, Howard University, 525 College Street NW, Washington, DC 20059, USA

## Abstract

In the title compound, C_17_H_24_NO_2_^+^·Br^−^·H_2_O, the pentyl group chain in the cation extends nearly perpendicular [N—C—C—C = −64.4 (3)°] to the mean plane of the indole ring with the carboxyl end group twisted such that the dihedral angle between the mean planes of the indole and carb­oxy groups measures 43.2 (4)°. Both ions in the salt form inter­molecular hydrogen bonds (O—H⋯Br and O—H⋯O) with the water mol­ecule. As a result of the Br⋯H—O—H⋯Br inter­actions, a zigzag chain is formed in the *c*-axis direction. The crystal packing is influenced by the collective action of the O—H⋯O and O—H⋯Br inter­molecular inter­actions as well as π–π stacking inter­molecular inter­actions between adjacent benzyl rings of the indole group [centroid–centroid distance = 3.721 (13) Å] and inter­molecular C—H⋯π inter­actions between a methyl hydrogen and the benzyl ring of the indole group. The O—H⋯Br inter­actions form a distorted tetra­hedral array about the central Br atom. A MOPAC AM1 calculation provides support to these observations.

## Related literature

For chemical and biological background, see: Zhu *et al.* (1994 ▶); Schwartz & Ulfelder (1992 ▶); Bengtsson *et al.* (2003 ▶); Hirons *et al.* (1994 ▶); Kurihara *et al.* (1977 ▶); Armitage & O’Brien (1992 ▶); Reers *et al.* (1991 ▶); Jung & Kim (2006 ▶); Menger & Pertusati (2008 ▶). A geometry optimized MOPAC AM1 computational calculation was performed using *WebMO Pro* (Schmidt & Polik, 2007 ▶).
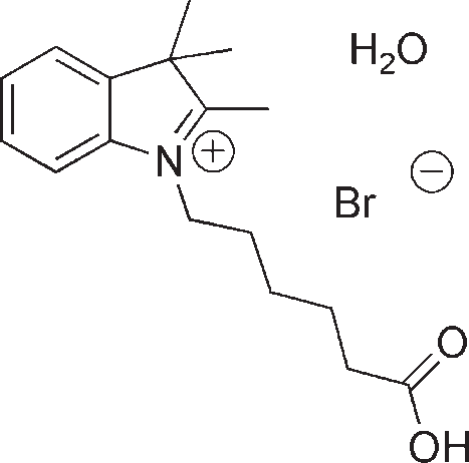


## Experimental

### 

#### Crystal data

C_17_H_24_NO_2_^+^·Br^−^·H_2_O
*M* *_r_* = 372.30
Monoclinic, 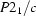

*a* = 14.4528 (3) Å
*b* = 15.3367 (2) Å
*c* = 8.0810 (2) Å
β = 99.437 (2)°
*V* = 1766.98 (6) Å^3^
*Z* = 4
Cu *K*α radiation
μ = 3.27 mm^−1^
*T* = 200 K
0.55 × 0.18 × 0.12 mm

#### Data collection

Oxford Diffraction Gemini R diffractometer
Absorption correction: multi-scan (*CrysAlis RED*; Oxford Diffraction, 2007 ▶) *T*_min_ = 0.296, *T*_max_ = 0.676
13155 measured reflections
3504 independent reflections
3049 reflections with *I* > 2σ(*I*)
*R*_int_ = 0.033

#### Refinement

*R*[*F* ^2^ > 2σ(*F* ^2^)] = 0.037
*wR*(*F* ^2^) = 0.098
*S* = 1.06
3504 reflections
209 parameters
3 restraints
H atoms treated by a mixture of independent and constrained refinement
Δρ_max_ = 0.52 e Å^−3^
Δρ_min_ = −0.43 e Å^−3^

### 

Data collection: *CrysAlis Pro* (Oxford Diffraction, 2007 ▶); cell refinement: *CrysAlis Pro*; data reduction: *CrysAlis Pro*; program(s) used to solve structure: *SHELXS97* (Sheldrick, 2008 ▶); program(s) used to refine structure: *SHELXL97* (Sheldrick, 2008 ▶); molecular graphics: *SHELXTL* (Sheldrick, 2008 ▶); software used to prepare material for publication: *SHELXTL*.

## Supplementary Material

Crystal structure: contains datablocks global, I. DOI: 10.1107/S1600536809049204/fl2265sup1.cif

Structure factors: contains datablocks I. DOI: 10.1107/S1600536809049204/fl2265Isup2.hkl

Additional supplementary materials:  crystallographic information; 3D view; checkCIF report

## Figures and Tables

**Table 1:** Hydrogen-bond geometry (Å, °)

*D*—H⋯*A*	*D*—H	H⋯*A*	*D*⋯*A*	*D*—H⋯*A*
O2—H2*O*⋯O1*W*	0.84	1.82	2.637 (4)	166
O1*W*—H1*W*1⋯Br	0.812 (19)	2.431 (19)	3.240 (2)	175 (5)
O1*W*—H1*W*2⋯Br^i^	0.817 (19)	2.47 (2)	3.262 (3)	165 (5)
C4—H4*B*⋯*Cg*2^i2^	0.99	2.88	3.828 (3)	162

Symmetry code: (i) 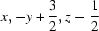; (ii) 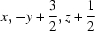. *Cg*2 is the centroid of the C6–C11 ring.

**Fig. 1.**
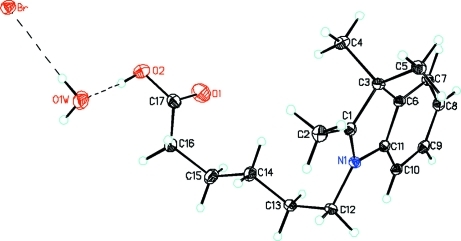
The molecular structure of (I), showing the atom numbering scheme and 50% probability displacement ellipsoids. Dashed lines show the O2–H2O···O1W and O1W–H1W1···Br hydrogen bonds.

**Fig. 2.**
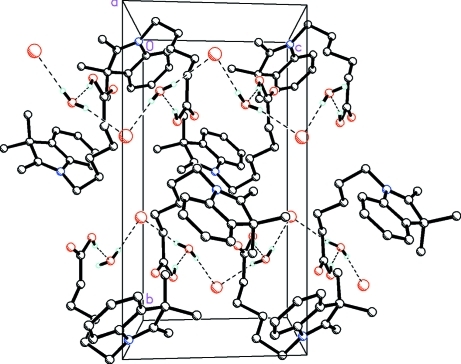
The molecular packing for (I) viewed down the a axis of the unit cell. Dashed lines indicate intermolecular O2–H2O···O1W, and O1W–H1W1···Br interactions. The O–H···Br···H–O interactions form a zigzag chain in the c direction.

**Fig. 3.**
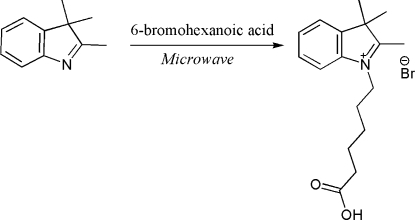
The formation of the title compound.

## supplementary crystallographic information

### Comment

The title compound,*C*_17_H_24_NO_2_^+^, Br^-^, H_2_O, (I), a salt with a crystallized water molecule, was synthesized under microwave conditions (Scheme 1). It has been used as a precursor for cyanine dyes which have widespread application as fluorescent probes. They have been used in DNA sequencing, immunoassays, agarose gel and capillary electrophoresis staining (Zhu *et al.*, (1994)), DNA analysis in polymerization chain reactions (Schwartz *et al.*, (1992); Bengtsson *et al.*, (2003)), in flow cytometry (Hirons *et al.*, (1994)), or as fluorescent probes for membrane fluidity (Kurihara *et al.*, 1977); Armitage *et al.*, 1992)) as well as in membrane potential studies (Reers *et al.*, (1991)). This precursor is of particular importance due to the presence of the carboxylic acid group, which when converted to the NHS ester, allows the attachment of these dyes to proteins.

In the cation, the mean plane of indole ring bisects the angle between the two attached 3,3 dimethyl groups (angles C1–C3–C4 = 109.1 (2)°; C1–C3–C5 = 111.86 (19)°; C5–C3–C4 = 110.3 (2)°) whereas the third methyl group is nearly in the plane of the indole ring (torsion angle C2–C1–C3–C6 = 178.4 (2)°), Fig. 1. The pentyl group chain extends nearly perpendicular to the mean plane of the indole ring (torsion angle N1–C12–C13–C14 = -64.4 (3)°) with the carboxyl end group twisted such that the dihedral angle between the mean planes of the indole and carboxy groups measures 43.2 (4)° (torsion angle C14–C15–C16–C17 = 94.4 (4)°).

Both ions from the salt form intermolecular hydrogen bonds (O–H···Br & O–H···O) with the water molecule. As a result of the Br···H–O–H···Br interactions a zigzag one-dimensional chain is formed in the c direction.(Fig. 2). Crystal packing is influenced by the collective action of intermolecular O—H···O and O—H···Br hydrogen bond interactions as well as π-π stacking intermolecular interactions between the center of gravity of nearby benzyl rings of the indole group (*Cg*2···*Cg*2: 3.721 (13) Å; slippage = 1.514 Å; 2 - *x*, 1 - *y*, 1 - *z*) and π-ring C4–H4B···*Cg*2 intermolecular interactions between a methyl hydrogen and the benzene ring of the indole group [H···*Cg* = 2.88 Å; *X*—H···*Cg* = 162°; *X*···CgX-H = 3.828 (3) Å; *Cg*2 = C6–C11; *x*, 3/2 - *y*, 1/2 + *z*] in the unit cell (Fig. 3). In addition there are weak C—H···Br interactions between the phenyl H atoms of two adjoining cations which, together with the O–H···Br interactions, form a distorted tetrahedral array about the central Br.

A geometry optimized MOPAC AM1 computational calculation was performed on the cation in the absence of the bromide ion and water molecule using *WebMO Pro* (Schmidt & Polik, 2007). The Hartree-Fock closed-shell (restricted) wavefunction along with [AM1 (Austin Model 1)] was used and minimizations were terminated at an r.m.s. gradient of less than 0.01 kJ mol^-1^ Å^-1^]. As a result of this calculation the dihedral angle between the mean planes of the indole and carboxy groups changes from 43.2 (4)° to 34.5 (2) Å. From this it is apparent that the collective influence of O–H···O and O–H···Br hydrogen bonds, weaker C–H···Br intermolecular interactions, π-π stacking intermolecular interactions, and π-ring C–H···*Cg*2 intermolecular interactions significantly influence crystal packing for this molecule.

### Experimental

The title compound (I) has been previously synthesized by refluxing reagents with the solvent *o*-dichloro-benzene for 12–24 h followed by filtration (Jung *et al.*, (2006); Menger *et al.* (2008)). For our study the title compound, (I), was synthesized as follows: 6-bromohexanoic acid (0.67 g, 0.0034 moles) and 2,3,3-trimethylindolenine (0.54 ml, 0.0034 moles) were added to a reaction vial *via* syringe and heated at 433 K for 1200 s and a ramp of 150 s in a Biotage Initiator microwave system (Scheme 2). The crystals were washed with acetone and dried under vacuum to yield (0.51 g, 42%). The sample was recrystallized by dissolving in dichloromethane then allowed to evaporate slowly at room temperature. ^1^H-NMR (DMSO-d_6_, 400 MHz): δ (p.p.m.): 7.87–8.01 (m, 1H), 7.78–7.87 (m, 1H), 7.26–7.64 (m, 2H) 4.45 (t, J = 7.6 Hz, 2H), 2.87 (s, 3H), 2.24 (t, J = 7.2 Hz, 2H), 1.85–1.81 (m, 2H), 1.6–1.5 (m, 8H), 1.44 (m, 3H); ^13^C-NMR (DMSO-d_6_, 100 MHz): δ (p.p.m.): 196.5 (C), 174.2 (C), 141.8 (C), 141.0 (C), 129.3 (CH), 128.9 (CH), 123.5 (CH), 115.4 (CH), 54.1 (C), 47.4 (CH_2_), 33.3 (CH_2_), 26.9 (CH_2_), 25.4 (CH_2_), 24.0 (CH_2_), 22.0 (CH_3_), 14.0 (CH_3_).

### Refinement

H1W1, H1W2 were obtained from a difference fourier map and refined with *U*_iso_(H) = 1.5*U*_eq_(O). The rest of the H atoms were placed in their calculated positions and then refined using a riding model with C(O)—H distances ranging from 0.84 to 0.99 Å, and with *U*_iso_(H) = 1.2_eq_(C,*O*) [1.5*U*_eq_ for CH_3_].

### Crystal data

**Table:**

C_17_H_24_NO_2_^+^·Br^−^·H_2_O	*F*(000) = 776
*M**_r_* = 372.30	*D*_x_ = 1.399 Mg m^−^^3^
Monoclinic, *P*2_1_/*c*	Cu *K*α radiation, λ = 1.54178 Å
Hall symbol: -P 2ybc	Cell parameters from 8515 reflections
*a* = 14.4528 (3) Å	θ = 5.6–73.5°
*b* = 15.3367 (2) Å	µ = 3.27 mm^−^^1^
*c* = 8.0810 (2) Å	*T* = 200 K
β = 99.437 (2)°	Needle, colorless
*V* = 1766.98 (6) Å^3^	0.55 × 0.18 × 0.12 mm
*Z* = 4	

### Data collection

**Table:**

Oxford Diffraction Gemini R diffractometer	3504 independent reflections
Radiation source: fine-focus sealed tube	3049 reflections with *I* > 2σ(*I*)
graphite	*R*_int_ = 0.033
Detector resolution: 10.5081 pixels mm^-1^	θ_max_ = 73.7°, θ_min_ = 5.8°
φ and ω scans	*h* = −17→15
Absorption correction: multi-scan (*CrysAlis RED*; Oxford Diffraction, 2007)	*k* = −18→19
*T*_min_ = 0.296, *T*_max_ = 0.676	*l* = −10→8
13155 measured reflections	

### Refinement

**Table:**

Refinement on *F*^2^	Primary atom site location: structure-invariant direct methods
Least-squares matrix: full	Secondary atom site location: difference Fourier map
*R*[*F*^2^ > 2σ(*F*^2^)] = 0.037	Hydrogen site location: inferred from neighbouring sites
*wR*(*F*^2^) = 0.098	H atoms treated by a mixture of independent and constrained refinement
*S* = 1.06	*w* = 1/[σ^2^(*F*_o_^2^) + (0.0518*P*)^2^ + 1.5238*P*] where *P* = (*F*_o_^2^ + 2*F*_c_^2^)/3
3504 reflections	(Δ/σ)_max_ = 0.001
209 parameters	Δρ_max_ = 0.52 e Å^−^^3^
3 restraints	Δρ_min_ = −0.43 e Å^−^^3^

### Special details

**Table:**

Geometry. All e.s.d.'s (except the e.s.d. in the dihedral angle between two l.s. planes) are estimated using the full covariance matrix. The cell e.s.d.'s are taken into account individually in the estimation of e.s.d.'s in distances, angles and torsion angles; correlations between e.s.d.'s in cell parameters are only used when they are defined by crystal symmetry. An approximate (isotropic) treatment of cell e.s.d.'s is used for estimating e.s.d.'s involving l.s. planes.
Refinement. Refinement of *F*^2^ against ALL reflections. The weighted *R*-factor *wR* and goodness of fit *S* are based on *F*^2^, conventional *R*-factors *R* are based on *F*, with *F* set to zero for negative *F*^2^. The threshold expression of *F*^2^ > σ(*F*^2^) is used only for calculating *R*-factors(gt) *etc*. and is not relevant to the choice of reflections for refinement. *R*-factors based on *F*^2^ are statistically about twice as large as those based on *F*, and *R*- factors based on ALL data will be even larger.

### Fractional atomic coordinates and isotropic or equivalent isotropic displacement parameters (Å^2^)

**Table:**

	*x*	*y*	*z*	*U*_iso_*/*U*_eq_	
Br	0.168343 (18)	0.871933 (16)	0.50535 (3)	0.03689 (11)	
O1	0.5587 (2)	0.7918 (2)	0.1347 (5)	0.0893 (10)	
O2	0.42824 (18)	0.81569 (14)	0.2331 (4)	0.0661 (7)	
H2O	0.3800	0.7873	0.2448	0.079*	
O1W	0.28626 (17)	0.7310 (2)	0.3254 (3)	0.0707 (8)	
H1W1	0.254 (3)	0.764 (3)	0.372 (5)	0.106*	
H1W2	0.251 (3)	0.714 (3)	0.242 (4)	0.106*	
N1	0.79026 (13)	0.52889 (12)	0.5047 (2)	0.0254 (4)	
C1	0.76946 (16)	0.56100 (15)	0.6432 (3)	0.0279 (5)	
C2	0.68861 (18)	0.53476 (18)	0.7221 (3)	0.0379 (6)	
H2A	0.6566	0.4853	0.6607	0.057*	
H2B	0.6449	0.5838	0.7192	0.057*	
H2C	0.7106	0.5178	0.8389	0.057*	
C3	0.84000 (16)	0.63015 (14)	0.7139 (3)	0.0268 (5)	
C4	0.78975 (19)	0.71914 (16)	0.7114 (3)	0.0367 (6)	
H4A	0.7579	0.7317	0.5972	0.055*	
H4B	0.8359	0.7649	0.7478	0.055*	
H4C	0.7435	0.7173	0.7875	0.055*	
C5	0.88798 (19)	0.60805 (17)	0.8929 (3)	0.0348 (5)	
H5A	0.9155	0.5496	0.8944	0.052*	
H5B	0.8416	0.6097	0.9687	0.052*	
H5C	0.9375	0.6508	0.9297	0.052*	
C6	0.90648 (16)	0.62789 (13)	0.5885 (3)	0.0252 (4)	
C7	0.98805 (16)	0.67357 (15)	0.5803 (3)	0.0301 (5)	
H7A	1.0118	0.7148	0.6643	0.036*	
C8	1.03446 (17)	0.65748 (16)	0.4452 (3)	0.0327 (5)	
H8A	1.0904	0.6886	0.4369	0.039*	
C9	1.00061 (17)	0.59686 (16)	0.3225 (3)	0.0318 (5)	
H9A	1.0338	0.5873	0.2319	0.038*	
C10	0.91882 (16)	0.54979 (15)	0.3296 (3)	0.0281 (5)	
H10A	0.8952	0.5078	0.2470	0.034*	
C11	0.87436 (15)	0.56811 (14)	0.4644 (3)	0.0246 (4)	
C12	0.73635 (17)	0.46508 (15)	0.3907 (3)	0.0320 (5)	
H12A	0.6940	0.4319	0.4522	0.038*	
H12B	0.7800	0.4232	0.3508	0.038*	
C13	0.67861 (17)	0.51091 (16)	0.2403 (3)	0.0339 (5)	
H13A	0.7215	0.5454	0.1823	0.041*	
H13B	0.6488	0.4661	0.1607	0.041*	
C14	0.60247 (18)	0.57112 (19)	0.2847 (3)	0.0391 (6)	
H14A	0.5631	0.5384	0.3527	0.047*	
H14B	0.6323	0.6199	0.3540	0.047*	
C15	0.5405 (2)	0.6079 (2)	0.1308 (4)	0.0553 (8)	
H15A	0.5107	0.5590	0.0617	0.066*	
H15B	0.5800	0.6403	0.0628	0.066*	
C16	0.46391 (19)	0.66849 (18)	0.1732 (4)	0.0430 (6)	
H16A	0.4067	0.6601	0.0888	0.052*	
H16B	0.4486	0.6516	0.2838	0.052*	
C17	0.4896 (2)	0.7628 (2)	0.1783 (4)	0.0492 (7)	

### Atomic displacement parameters (Å^2^)

**Table:**

	*U*^11^	*U*^22^	*U*^33^	*U*^12^	*U*^13^	*U*^23^
Br	0.04080 (17)	0.03133 (16)	0.03975 (18)	−0.00006 (10)	0.01018 (12)	0.00063 (10)
O1	0.0628 (16)	0.0726 (18)	0.136 (3)	−0.0271 (14)	0.0274 (17)	−0.0028 (18)
O2	0.0607 (14)	0.0372 (11)	0.097 (2)	0.0081 (10)	0.0028 (13)	−0.0153 (12)
O1W	0.0458 (12)	0.102 (2)	0.0635 (16)	0.0124 (13)	0.0056 (11)	−0.0374 (15)
N1	0.0266 (9)	0.0221 (8)	0.0263 (10)	−0.0014 (7)	0.0009 (7)	0.0013 (7)
C1	0.0300 (11)	0.0264 (11)	0.0259 (11)	0.0015 (9)	0.0005 (9)	0.0053 (9)
C2	0.0355 (13)	0.0415 (14)	0.0380 (14)	−0.0016 (10)	0.0105 (11)	0.0069 (11)
C3	0.0325 (11)	0.0237 (11)	0.0238 (11)	−0.0012 (8)	0.0033 (9)	−0.0004 (8)
C4	0.0450 (14)	0.0296 (12)	0.0366 (14)	0.0056 (10)	0.0100 (11)	−0.0002 (10)
C5	0.0439 (14)	0.0343 (12)	0.0244 (12)	−0.0008 (10)	0.0004 (10)	0.0014 (10)
C6	0.0317 (11)	0.0205 (10)	0.0225 (11)	0.0018 (8)	0.0015 (9)	0.0015 (8)
C7	0.0334 (11)	0.0232 (11)	0.0319 (13)	−0.0025 (9)	0.0003 (9)	0.0005 (9)
C8	0.0302 (11)	0.0282 (11)	0.0394 (14)	−0.0014 (9)	0.0051 (10)	0.0082 (10)
C9	0.0348 (12)	0.0313 (12)	0.0306 (13)	0.0075 (9)	0.0089 (10)	0.0065 (10)
C10	0.0347 (12)	0.0244 (11)	0.0239 (11)	0.0050 (9)	0.0010 (9)	0.0006 (9)
C11	0.0275 (10)	0.0210 (10)	0.0243 (11)	0.0008 (8)	0.0015 (8)	0.0045 (8)
C12	0.0327 (11)	0.0234 (11)	0.0374 (13)	−0.0030 (9)	−0.0020 (10)	−0.0040 (10)
C13	0.0330 (12)	0.0330 (12)	0.0332 (13)	0.0007 (9)	−0.0018 (10)	−0.0069 (10)
C14	0.0352 (13)	0.0426 (14)	0.0381 (14)	0.0067 (11)	0.0023 (11)	−0.0017 (11)
C15	0.0538 (18)	0.0570 (18)	0.0493 (18)	0.0244 (15)	−0.0087 (14)	−0.0133 (15)
C16	0.0363 (13)	0.0373 (14)	0.0526 (17)	0.0073 (11)	−0.0014 (12)	−0.0026 (12)
C17	0.0405 (15)	0.0418 (15)	0.062 (2)	−0.0031 (12)	−0.0019 (13)	−0.0028 (14)

### Geometric parameters (Å, °)

**Table:**

O1—C17	1.198 (4)	C7—C8	1.394 (4)
O2—C17	1.330 (4)	C7—H7A	0.9500
O2—H2O	0.8400	C8—C9	1.389 (4)
O1W—H1W1	0.812 (19)	C8—H8A	0.9500
O1W—H1W2	0.817 (19)	C9—C10	1.394 (3)
N1—C1	1.302 (3)	C9—H9A	0.9500
N1—C11	1.440 (3)	C10—C11	1.381 (3)
N1—C12	1.476 (3)	C10—H10A	0.9500
C1—C2	1.476 (3)	C12—C13	1.528 (3)
C1—C3	1.516 (3)	C12—H12A	0.9900
C2—H2A	0.9800	C12—H12B	0.9900
C2—H2B	0.9800	C13—C14	1.524 (3)
C2—H2C	0.9800	C13—H13A	0.9900
C3—C6	1.507 (3)	C13—H13B	0.9900
C3—C5	1.536 (3)	C14—C15	1.518 (4)
C3—C4	1.545 (3)	C14—H14A	0.9900
C4—H4A	0.9800	C14—H14B	0.9900
C4—H4B	0.9800	C15—C16	1.526 (4)
C4—H4C	0.9800	C15—H15A	0.9900
C5—H5A	0.9800	C15—H15B	0.9900
C5—H5B	0.9800	C16—C17	1.493 (4)
C5—H5C	0.9800	C16—H16A	0.9900
C6—C11	1.382 (3)	C16—H16B	0.9900
C6—C7	1.382 (3)		
			
C17—O2—H2O	109.5	C8—C9—H9A	119.4
H1W1—O1W—H1W2	104 (3)	C10—C9—H9A	119.4
C1—N1—C11	111.00 (19)	C11—C10—C9	115.8 (2)
C1—N1—C12	127.9 (2)	C11—C10—H10A	122.1
C11—N1—C12	120.96 (19)	C9—C10—H10A	122.1
N1—C1—C2	125.4 (2)	C10—C11—C6	124.3 (2)
N1—C1—C3	110.6 (2)	C10—C11—N1	127.7 (2)
C2—C1—C3	124.0 (2)	C6—C11—N1	108.0 (2)
C1—C2—H2A	109.5	N1—C12—C13	110.80 (19)
C1—C2—H2B	109.5	N1—C12—H12A	109.5
H2A—C2—H2B	109.5	C13—C12—H12A	109.5
C1—C2—H2C	109.5	N1—C12—H12B	109.5
H2A—C2—H2C	109.5	C13—C12—H12B	109.5
H2B—C2—H2C	109.5	H12A—C12—H12B	108.1
C6—C3—C1	101.18 (18)	C14—C13—C12	114.4 (2)
C6—C3—C5	112.9 (2)	C14—C13—H13A	108.7
C1—C3—C5	111.86 (19)	C12—C13—H13A	108.7
C6—C3—C4	111.20 (19)	C14—C13—H13B	108.7
C1—C3—C4	109.1 (2)	C12—C13—H13B	108.7
C5—C3—C4	110.3 (2)	H13A—C13—H13B	107.6
C3—C4—H4A	109.5	C15—C14—C13	112.6 (2)
C3—C4—H4B	109.5	C15—C14—H14A	109.1
H4A—C4—H4B	109.5	C13—C14—H14A	109.1
C3—C4—H4C	109.5	C15—C14—H14B	109.1
H4A—C4—H4C	109.5	C13—C14—H14B	109.1
H4B—C4—H4C	109.5	H14A—C14—H14B	107.8
C3—C5—H5A	109.5	C14—C15—C16	113.2 (3)
C3—C5—H5B	109.5	C14—C15—H15A	108.9
H5A—C5—H5B	109.5	C16—C15—H15A	108.9
C3—C5—H5C	109.5	C14—C15—H15B	108.9
H5A—C5—H5C	109.5	C16—C15—H15B	108.9
H5B—C5—H5C	109.5	H15A—C15—H15B	107.7
C11—C6—C7	119.3 (2)	C17—C16—C15	114.2 (3)
C11—C6—C3	109.15 (19)	C17—C16—H16A	108.7
C7—C6—C3	131.6 (2)	C15—C16—H16A	108.7
C6—C7—C8	118.1 (2)	C17—C16—H16B	108.7
C6—C7—H7A	120.9	C15—C16—H16B	108.7
C8—C7—H7A	120.9	H16A—C16—H16B	107.6
C9—C8—C7	121.3 (2)	O1—C17—O2	120.4 (3)
C9—C8—H8A	119.4	O1—C17—C16	124.5 (3)
C7—C8—H8A	119.4	O2—C17—C16	115.1 (3)
C8—C9—C10	121.3 (2)		
			
C11—N1—C1—C2	−179.2 (2)	C8—C9—C10—C11	0.5 (3)
C12—N1—C1—C2	4.5 (4)	C9—C10—C11—C6	−0.7 (3)
C11—N1—C1—C3	1.0 (2)	C9—C10—C11—N1	−179.0 (2)
C12—N1—C1—C3	−175.3 (2)	C7—C6—C11—C10	0.2 (3)
N1—C1—C3—C6	−1.7 (2)	C3—C6—C11—C10	−180.0 (2)
C2—C1—C3—C6	178.4 (2)	C7—C6—C11—N1	178.82 (19)
N1—C1—C3—C5	−122.2 (2)	C3—C6—C11—N1	−1.4 (2)
C2—C1—C3—C5	58.0 (3)	C1—N1—C11—C10	178.8 (2)
N1—C1—C3—C4	115.6 (2)	C12—N1—C11—C10	−4.6 (3)
C2—C1—C3—C4	−64.3 (3)	C1—N1—C11—C6	0.3 (2)
C1—C3—C6—C11	1.9 (2)	C12—N1—C11—C6	176.90 (19)
C5—C3—C6—C11	121.6 (2)	C1—N1—C12—C13	99.2 (3)
C4—C3—C6—C11	−113.9 (2)	C11—N1—C12—C13	−76.8 (3)
C1—C3—C6—C7	−178.4 (2)	N1—C12—C13—C14	−64.4 (3)
C5—C3—C6—C7	−58.7 (3)	C12—C13—C14—C15	−174.0 (2)
C4—C3—C6—C7	65.8 (3)	C13—C14—C15—C16	−179.9 (3)
C11—C6—C7—C8	0.4 (3)	C14—C15—C16—C17	94.4 (4)
C3—C6—C7—C8	−179.3 (2)	C15—C16—C17—O1	8.5 (5)
C6—C7—C8—C9	−0.5 (4)	C15—C16—C17—O2	−172.8 (3)
C7—C8—C9—C10	0.0 (4)		

### Hydrogen-bond geometry (Å, °)

**Table:**

*D*—H···*A*	*D*—H	H···*A*	*D*···*A*	*D*—H···*A*
O2—H2O···O1W	0.84	1.82	2.637 (4)	166
O1W—H1W1···Br	0.81 (2)	2.43 (2)	3.240 (2)	175 (5)
O1W—H1W2···Br^i^	0.82 (2)	2.47 (2)	3.262 (3)	165 (5)
C4—H4B···Cg2^ii^	0.99	2.88	3.828 (3)	162

Symmetry codes: (i) *x*, −*y*+3/2, *z*−1/2; (ii) *x*, −*y*+3/2, *z*+1/2.
